# Inappropriate prescribing in geriatric rural primary care: impact on adverse outcomes and relevant risk factors in a prospective observational cohort study

**DOI:** 10.1007/s40520-023-02475-y

**Published:** 2023-07-10

**Authors:** Maria Tampaki, Alexandra Livada, Maria-Niki Fourka, Elli Lazaridou, Marina Kotsani, Athanase Benetos, Petros P. Sfikakis, Evrydiki Kravvariti

**Affiliations:** 1 Postgraduate Medical Studies in Geriatric Syndromes and Physiology of Aging, School of Medicine, National and Kapodistrian University of Athens, Athens, Greece; 2 Department of Statistics, Athens University of Economics and Business, Athens, Greece; 3 Primary Medical Care Unit of Marmari, S. Evia, General Hospital of Karystos, Karystos, Greece; 4 Pôle « Maladies du Vieillissement, Gérontologie Et Soins Palliatifs », Université de Lorraine, CHRU-Nancy, Nancy, France; 5 Pôle « Maladies du Vieillissement, Gérontologie Et Soins Palliatifs », and INSERM DCAC u1116, Université de Lorraine, CHRU-Nancy, 54000 Nancy, France; 6 First Department of Propaedeutic and Internal Medicine, Joint Academic Rheumatology Program, School of Medicine, National and Kapodistrian University of Athens, 75, Mikras Asias St., Goudi, 11527 Athens, Greece; 7 Hellenic Society for the Study and Research of Aging, Athens, Greece

**Keywords:** Adverse drug outcomes, Geriatric polypharmacy, Inappropriate prescribing, Risk factors, START/STOPP criteria

## Abstract

**Background:**

Several tools have revealed an association between potentially inappropriate medications (PIM) and adverse outcomes, but the one most fitted for the rural population has not been determined.

**Aims:**

We investigated the performance of the Screening Tool of Older Persons' Prescriptions (STOPP) and Screening Tool to Alert doctors to the Right Treatment (START) in identifying inappropriate prescribing and its association with adverse outcomes among older rural primary health care users.

**Methods:**

A cohort of consenting outpatients aged ≥ 65 years in a rural Greek primary care center was assessed for PIM and potential prescribing omissions (PPO) using the START/STOPP version 2 criteria. Medications, comorbidities, functional status, and laboratory data were recorded along with 6-month incidence of emergency department visits, hospitalization, and death prospectively.

**Results:**

Among 104 participants (median age 78 years, 49.1% women, receiving a median of 6 drugs), PPO was found in 78% and PIMs in 61%. PIM was multivariately correlated with multimorbidity (*p* = 0.029) and polypharmacy (*p* < 0,001), while drug-PPO was only associated with multimorbidity (*p* = 0.039). The number of PIM predicted emergency department visits and hospitalizations at 6-month follow-up (*p* value 0.011), independent of age, sex, frailty, comorbidities, and total medication number.

**Discussion:**

The START/STOPP tool is useful in identifying inappropriate prescribing patterns leading to increased utilization of acute care services in older adults followed at a rural primary care setting.

**Conclusion:**

Inappropriate prescribing as identified by the START/STOPP criteria is prevalent among older adults with multimorbidity in rural primary care, and independently associated with future acute care visits.

**Supplementary Information:**

The online version contains supplementary material available at 10.1007/s40520-023-02475-y.

## Introduction

Μultimorbidity in the older adult population has led to increasingly complex drug regimens, potentially harmful polypharmacy [1], and poor treatment adherence [2]. Geriatric research has revealed that most adverse outcomes attributable to polypharmacy are related to potentially inappropriate medications (PIM), which are more common among women rather than men, older rather than younger patients, and those with impaired autonomy in daily activities, a high overall number of comorbidities and drugs [3, 4]. In addition, PIM in older adults have been associated with numerous geriatric syndromes and negative health outcomes [5], increased use of health services, more frequent hospitalizations, and consequently higher health costs [6] as well as higher mortality [7, 8], highlighting the need of using prescribing tools [9, 10]; however, other cohorts have yielded conflicting results and did not confirm increased mortality [11, 12]. In addition, the optimal tool to evaluate inappropriate prescribing has not been determined.

Several validated screening tools have been developed to detect PIM in older adults, such as the Beers criteria [13], and the Screening Tool of Older People’s Prescriptions (STOPP) and criteria for potential prescribing omissions (PPO) called the Screening Tool to Alert to Right Treatment (START) developed in 2008 and updated in 2015 and 2018 [14]. According to a prospective cohort study, when comparing different tools, associations between inappropriate prescribing and outcomes differ: several prescribing tools have been associated with an increased rate of health system visits, while only STOPP was significantly associated with increased emergency attendance [15, 16]. Additionally, the STOPP/START tool includes prescribing omissions, and seems to identify more instances of potential major clinical relevance [17]. Although PIMs as defined by the STOPP/START criteria have been linked to hospitalizations and mortality in urban settings [6, 8, 11, 18], here is a paucity of data on PIMs-associated adverse outcomes in rural community settings.

In this study, we sought to identify risk factors associated with inappropriate prescribing as per the STOPP / START v2 in a rural health center in Greece, and prospectively assess increases in risk for emergency department visits, hospitalizations or death, in this population.

## Methods

### Study design, setting, and participants

A prospective observational cohort study of 104 consecutive home-dwelling outpatients aged ≥ 65 years was conducted from February to October 2019 in a rural Greek primary care center. The state regional medical center of Marmari, S. Evoia where our study took place serves a reference population of approximately 1000 people, of which about 30% are older adults. The study was approved by “Diocleion” General Hospital of Karystos institutional review board, and all participants signed written informed consent in accordance with the declaration of Helsinki and the European General Data Protection Regulation.

Demographics, regular medications (counted as active ingredients per person)—excluding over the counter medications—vaccination status, comorbidities as mentioned in revised START/STOPP criteria, functional status (using KATZ Index tool), frailty status (using the 9-point Clinical Frailty Scale) [19], multimorbidity (using the Charlson comorbidity index (CCI) [20]), laboratory values and renal function measured by glomerular filtration rate (GFR) were recorded by a geriatric expert, during the medical visit or in case of severely frail patients through representation by their caregivers in their encounter with the primary healthcare setting. The data were obtained through self-report, review of patient’s and reference hospital’s medical records, and complemented by the Greek universal electronic prescribing system. Participants were assessed by the investigator (MT) case by case for potentially inappropriate prescribing (PIP), including PIM and PPO using the START/STOPP v 2 criteria: the total number of potentially inappropriate medication applying STOPP criteria was regarded as PIM number. Regarding PPOs, we considered inadequate prescription practices applying START criteria in total, termed “PPO” (including drugs and/or vaccinations), and separately, drug prescribing omissions (regardless of vaccination omissions), termed “dPPO”, since vaccination omission is to a greater extent affected by personal attitudes and choices.

Six-month prospective outcomes were collected via telephone or in-person follow-up visits and/or electronic medical record review including deaths, emergency department visits, and hospitalizations.

### Statistical analysis

Age, Katz index, CFS, CCI, as well as number of medication and PIM number were regarded continuous variables. Sex, hospitalization/death, hospitalization/emergency department visit, PIM, PPO, and dPPO were treated as categoric variables. Data preparation and analysis were done in R (R Core Team, 2016). To investigate the associations between binary variables, Chi-square independence tests were performed and, wherever deemed necessary due to the sparse distribution of contingencies, Fisher's exact test was applied. To test if the total number of drugs was univariately associated with inappropriate prescribing and worse clinical outcomes, a non-parametric Kruskal–Wallis population difference test was performed. Finally, we further investigated the risk factors for PIM and dPPO (as binary variables) through multiple logistic regression models, including age, sex, CCI, CFS, and number of medications. The potential PIM risk factors identified in the relevant literature [21–23] were included a priori into multivariate models. Statistical significance was set at *α* = 0.05.

## Results

### Population characteristics

From a reference population of 1000 inhabitants, 140 adults older than 65 years presented to our medical center for an ambulatory primary care visit. Of those, 104 agreed to participate and were enrolled in the study. Median age was 78 years (interquartile range: 71.5–83.5 years), of which 51 (49.1%) were women. Half of the participants were in very good physical condition or coping well with everyday activities (CFS 1–3). Only three of them suffered from documented dementia. Baseline characteristics are shown in Table 1.

**Table 1:** Main clinical and demographic characteristics of the study participants (*N* = 104)

Median age, years (IQR)	78 (67–90)
Female sex, *n* (%)	51 (49%)
Median frailty status (IQR)	4 (1–7)
Fit (CFS 1–3), *n* (%)	51 (49%)
Intermediately frail (CFS 4–6), *n* (%)	48 (47%)
Severely frail–terminally ill (CFS 7–9), *n* (%)	5 (5%)
Median functional status	6
Completely autonomous in ADL (score 6), *n* (%)	86 (83%)
Partially assisted in ADL (score 3–5), *n* (%)	15 (14%)
Dependent in ADL (score 0–2), *n* (%)	3 (3%)
Median CCI (IQR)	1 (0–1)
Disease prevalence	
Hypertension	80%
Cardiovascular disease other than hypertension	71%
Endocrine disorders (mainly DM)	26%
CNS diseases	25%
Chronic kidney disease	15%
Respiratory tract diseases	13%
Urogenital disorders (mainly BPH, incontinence)	13%
Musculoskeletal disorders	12%
GI tract diseases	4%
Dementia	3%
Median no of drugs (IQR)	6 (1–11)
Polypharmacy (drugs ≥ 5), *n* (%)	70 (67%)
Median no of drugs for those with ≥ 5 drugs (IQR)	8 (4–12)
Prevalence of most prescribed drugs	
Statins	54%
PPIs	34%
Metformin	20%
Benzos	19%
XOIs	17%
SSRIs	15%
Antipsychotics	3%
NSAIDs	1%
Patients with at least 1 PIP, *n* (%)	99 (95%)
Patients with at least 1 PIM, *n* (%)	63 (61%)
Patients with at least 1 PPO, *n* (%)	81 (78%)
Among PPOs drug omission, *n* (%)	31 (38%)
Among PPOs vaccination omission, *n* (%)	68 (84%)
6-month adverse outcomes of interest	
Hospitalization, *n* (%)	19 (18%)
Falls, *n* (%)	5 (5%)
Delirium, *n* (%)	2 (2%)
Deaths, *n* (%)	4 (4%)
Emergency care visits, *n* (%)	23 (22%)
Composite of hospitalization and/or emergency care visit (termed “acute care visits”), *n* (%)	31 (30%)

*IQR* interquartile range, *CFS* clinical frailty scale, *ADL* activities of daily living, *CCI* Charlson comorbidity index, *GI* gastrointestinal, *DM* diabetes mellitus, *CNS* central nervous system, *BPH* benign prostate hyperplasia, *PPIs* proton pump inhibitors, *Benzos* benzodiazepines, *XOIs* xanthine oxidase inhibitors, *SSRI* selective serotonin reuptake inhibitors, *NSAIDs* non-steroid anti-inflammatory drugs, *PIP* potentially inappropriate prescribing, *PIM* potentially inappropriate medication, *PPO* potentially prescribing omission

In 95% of the sample, at least one criterion of PIP was met, with at least one PIM being observed in 61% of participants, while 78% of the cases concerning PPOs with major representative omission of vaccination in 84% of them.

In more detail, among those with PIM, the most frequent cause of inappropriateness was drug prescription beyond the recommended duration in 51%, followed by drug prescription without an evidence-based clinical indication (48% of PIM), duplicate drug class prescription in 14% of PIMs, and the prescription of drugs to treat side effects of other drugs (3%). Not impressively 100% of prescribed benzodiazepines were administered beyond recommended 4-week duration, while xanthine oxidase inhibitors were prescribed in 99% of cases without an evidence-based clinical indication. Similarly, proton pump inhibitors (PPIs') were prescribed either prolonged or without a documented clinical indication in half of the cases. (More details in Online Resource 1).

Among patients with PPOs, the vast majority regarded vaccination omission (84% of PPO). Drug omissions were noticed in 38% of PPOs with main representatives being lack of statin for secondary prevention in 7%, b-blockers in ischemic heart disease (5%), bone anti-resorptive or anabolic therapy in documented osteoporosis in 5%, followed by bisphosphonates/ vitamin D/ calcium in patients taking long-term systemic corticosteroid therapy in 4% of cases. (More details in Online Resource 2).

### Exploratory analysis

As expected, the number of PIMs was univariately associated to a statistically significant degree with patient age, the number of comorbidities (as per the CCI), and the level of frailty (as per the CFS), as well as with the total number of medications (Online Resources 3–4). All of the univariately associated factors were then included in the same multivariate regression models, to derive a fully adjusted analysis of PIM determinants and predictive value.

### Determinants of inappropriate prescribing

In multiple logistic regression models, PIM and dPPO were independently associated with the total comorbidity burden (CCI); (OR = 0.51, 95% CI = 0.28–0.93 and OR = 1.74, 95% CI = 1.03–2.94 respectively), while adjusting for age, sex, CCI, CFS, and the total medication number (Tables 2 and 3). PIMs, but not dPPOs, were also associated with the total medication number (OR = 1.57, 95% CI: 1.25–1.96). (Tables 2, 3).

**Table 2:** Multiple logistic regression model investigating determinants of potentially inappropriate medication in the study cohort

Variable	OR	95% CI	*p* value
Age	0.99	0.91–1.06	0.705
Female sex	1.82	0.71–4.71	0.215
CCI	0.51	0.28–0.93	0.029
Clinical frailty scale	1.35	0.92–2.00	0.126
Nr of meds	1.57	1.25–1.96	< 0.001
Age	0.99	0.91–1.06	0.705

*OR* odds ratio, *CI* confidence interval, *CCI* Charlson comorbidity index, *Nr of meds* number of medications

Underlined values identify statistically significant results, statistical significance is set at α = 0.05

**Table 3:** Multiple logistic regression model investigating determinants of drug prescribing omissions in the study cohort

Variable	OR	95% CI	p value
Age	1.05	0.97–1.127	0.199
Female sex	1.76	0.70–4.407	0.213
CCI	1.74	1.03–2.937	0.039
Clinical frailty scale	0.82	0.57–1.191	0.298
Nr of meds	1.00	0.85–1.163	0.946

*OR* odds ratio, *CI* confidence interval, *CCI* Charlson comorbidity index, *Nr of meds* number of medications

Underlined values identify statistically significant results, statistical significance is set at α = 0.05

### Prospective 6-month adverse outcomes

Four patients died during the 6-month follow-up (78, 84, 88, and 89 years old). Overall, 31 (29.8%) visited the hospital for an emergency department visit and/or unscheduled admission (composite outcome acute care visits). In more detail, 22.1% needed emergency medical services, 18.3% have been hospitalized.

**Fig. 1:**
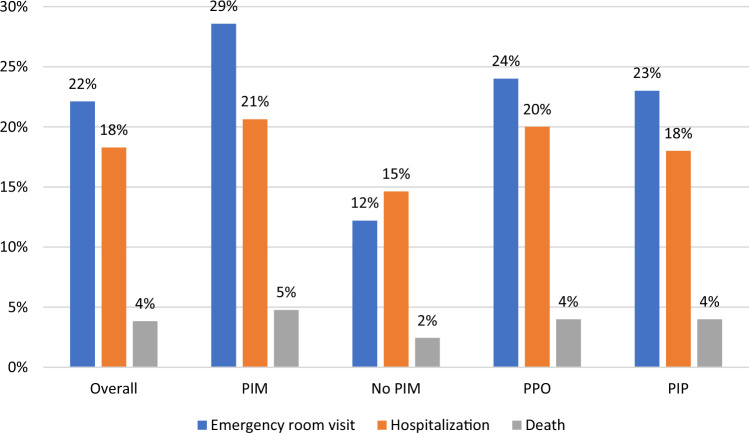
Prevalence of adverse outcomes (six-month incidence of emergency department visits, hospitalizations, and death) in the overall cohort (*N* = 104), among participants with potentially inappropriate medication (PIM, *N* = 63), among those without inappropriate medication (no PIM, *N* = 41), among those with potential prescribing omissions (PPO, *N* = 81) and among those with potentially inappropriate prescribing (either PIM or PPO) in general (PIP, *N* = 99)

PIM was statistically significantly associated with increased emergency department visits (*p* value 0.027), hospitalizations (*p* value 0.032), and deaths (*p* value 0.041) (Fig. 1, more details in Online Resources 5 and 6); these associations were robust to confounding effects in multiple logistic regression models adjusted for number of medications, age, sex, comorbidities, CFS and total number of medication, where also an independent association between acute care visits and older age was shown (Table 4). There was no univariate or multivariable association between dPPO and the 6-month outcomes of interest.

**Table 4:** Multiple logistic regression model investigating determinants of acute care visits (emergency department visits and/or hospitalizations) at 6 months in the study cohort

Variable	OR	95% CI	*p* value
Age	1.08	1.00–1.17	0.049
Sex	1.09	0.39–3.01	0.868
CCI	1.67	0.95–2.93	0.075
Clinical frailty scale	0.88	0.59–1.29	0.503
Nr of PIMS	2.06	1.18–3.57	0.011
Nr of meds	0.90	0.73–1.12	0.342

*OR* odds ratio, *CI* confidence interval, *CCI* Charlson comorbidity index, *Nr of meds* number of medications, *PIM* potentially inappropriate prescribing

Underlined values identify statistically significant results, statistical significance is set at α = 0.05

## Discussion

In our study at least one PIM recorded in 61% of participants, and at least one PPO in 78% of individuals. The corresponding prevalence of PIM recorded in the literature varies from 21% to 66.8% [12]. Only four studies in eastern Europe have evaluated the prevalence of inappropriate prescribing in primary care, of which three were held in urban hospitals [4, 24–26]; PIM prevalence was similar to our study in Croatia (69%, [24]), Albania (63%, [25]), and Italy (54%, [4]). Only one study was held in a rural setting in Romania [26], which found lower prevalence of PIP (26% PIM and 42% PPO); however, this study only applied a limited subset of the START/STOPP criteria. Notably, these estimates are much higher than recorded in Ireland (21% PIM and 23% PPOs) [27].. Heterogeneity of prevalence in various studies seems to be depended on variable primary care models, prescribing systems, and prescribing mentality among different countries, characteristics of the population studied as well as the tool used to capture inappropriate prescribing [12].

Several factors contribute to prescribing inertia in primary care in Greece and other European countries: lack of time, lack of information sharing among primary care practitioners and specialists, lack of structured special geriatric education [28, 29], inadequate numbers of health workforce and inadequate financial motivation [30]. It should also be noted that part of our data was based on self-report of the participants, so it is possible that patients may have failed to mention certain medical conditions, leading to a false identification of STOPP PIMs. However, multiple characteristics of the participants were tested, reducing the likelihood of systematic reference bias between subgroups.

Our study confirms a strong association between PIM and polypharmacy, which has been reported in previous studies [31–36], which, however, did not examine rural community-dwelling older adults using the START/STOPP screening tool, as in our work. We found that both PIM and PPO were directly related to the burden of comorbidities, which remains in keeping with previous studies assessing multiple prescribing tools and indices comorbidity [31, 32]; again, none of these studies was held exclusively in rural population. PPOs, in particular, have been associated to multimorbidity in a few studies held in hospitalized patients, but not in a rural community setting [4, 37].

Regarding the risk for future adverse events conferred by PIMs, studies on this topic are fraught by heterogeneous methodology on cardinal aspects such as the origin of patients (hospital, long-term care facilities, community), the classification tool for PIM (Beers criteria, START/STOPP, other screening methods), and the categorization of outcomes (use of health services, hospitalizations, length of hospital stay, death, health costs, heterogeneous quality of life criteria) [6]. The impact of PIM on acute care visits was documented in a systematic review by Hyttinen et al., while in other studies, inappropriate prescribing was associated with mortality [7, 8]. In a meta-analysis including 77,624 primary care participants, a significant association between PIP and increased emergency department visits and hospitalizations was found [11]; however, a very high degree of heterogeneity was noted, and all studies were conducted in urban primary care centers. Our study confirms that the elevated risk of acute care visits deriving from PIM prescribing is also relevant to the primary care of rural populations. We have also highlighted that a high burden of comorbidities, but not polypharmacy per se, is associated with prescribing omissions, which has not been described in such a population.

To our knowledge, this is the first study evaluating inappropriate prescribing in primary care in Greece, and one of the few referring to Southeastern Europe. Its strengths lie in the prospective design, detailed recording and classification of prescription patterns, and case by case evaluation for START/STOPP criteria ascertainment by a geriatric expert. A limitation of the study is the modest sample size, resulting in few cases experiencing adverse outcomes; larger data from additional rural primary care models are needed for more robust conclusion.

Our findings highlight the significance of using dedicated criteria to reinforce appropriate geriatric prescribing across primary care settings, including rural areas. Further research is needed to reveal the most appropriate method of integrating the STOPP / START tool into routine clinical practice and document its effectiveness in preventing suboptimal outcomes.

## Supplementary Information

Below is the link to the electronic supplementary material.

Supplementary file1 (DOCX 65 KB)

## References

[1] Guthrie B, Makubate B, Hernandez-Santiago V, Dreischulte T (2015). The rising tide of polypharmacy and drug-drug interactions: population database analysis 1995–2010. BMC Med.

[2] Ulley J, Harrop D, Ali A, Alton S, Fowler Davis S (2019). Deprescribing interventions and their impact on medication adherence in community-dwelling older adults with polypharmacy: a systematic review. BMC Geriatr..

[3] Pérez T, Moriarty F, Wallace E, McDowell R, Redmond P, Fahey T (2018). Prevalence of potentially inappropriate prescribing in older people in primary care and its association with hospital admission: longitudinal study. BMJ..

[4] Bo M, Gibello M, Brunetti E, Boietti E, Sappa M, Falcone Y, Aurucci ML, Iacovino M, Fonte G, Cappa G (2019). Prevalence and predictors of inappropriate prescribing according to the screening tool of older people’s prescriptions and screening tool to alert to right treatment version 2 criteria in older patients discharged from geriatric and internal medicine ward: STOPP/STARTv2 in hospital-discharged patients. Geriatr Gerontol Int.

[5] Mangin D, Bahat G, Golomb BA, Mallery LH, Moorhouse P, Onder G, Petrovic M, Garfinkel D (2018). International group for reducing inappropriate medication use & polypharmacy (IGRIMUP): position statement and 10 recommendations for action. Drugs Aging.

[6] Hyttinen V, Jyrkkä J, Valtonen H (2016). A systematic review of the impact of potentially inappropriate medication on health care utilization and costs among older adults. Med Care.

[7] Thomas RE, Nguyen LT, Jackson D, Naugler C (2020). Potentially inappropriate prescribing and potential prescribing omissions in 82,935 older hospitalised adults: association with hospital readmission and mortality within six months. Geriatrics..

[8] Cardwell K, Kerse N, Hughes CM, Teh R, Moyes SA, Menzies O, Rolleston A, Broad JB, Ryan C (2020). Does potentially inappropriate prescribing predict an increased risk of admission to hospital and mortality? A longitudinal study of the “oldest old”. BMC Geriatr.

[9] Gibert P, Cabaret M, Moulis M, Bosson J-L, Boivin J-E, Chanoine S, Allenet B, Bedouch P, Gavazzi G (2018). Optimizing medication use in elderly people in primary care: Impact of STOPP criteria on inappropriate prescriptions. Arch Gerontol Geriatr.

[10] Liew TM, Lee CS, Goh SKL, Chang ZY (2020). The prevalence and impact of potentially inappropriate prescribing among older persons in primary care settings: multilevel meta-analysis. Age Ageing.

[11] Liew TM, Lee CS, Goh Shawn KL, Chang ZY (2019). Potentially inappropriate prescribing among older persons: a meta-analysis of observational studies. Ann Fam Med..

[12] Hill-Taylor B, Walsh KA, Stewart S, Hayden J, Byrne S, Sketris IS (2016). Effectiveness of the STOPP/START (screening tool of older persons’ potentially inappropriate prescriptions/screening tool to alert doctors to the right treatment) criteria: systematic review and meta-analysis of randomized controlled studies. J Clin Pharm Ther.

[13] By the 2019 American Geriatrics Society Beers Criteria® Update Expert Panel, American Geriatrics Society 2019 Updated AGS Beers Criteria® for Potentially Inappropriate Medication Use in Older Adults, J Am Geriatr Soc. 67 (2019) 674–694. 10.1111/jgs.15767 10.1111/jgs.15767 30693946

[14] O’Mahony D, O’Sullivan D, Byrne S, O’Connor MN, Ryan C, Gallagher P (2015). STOPP/START criteria for potentially inappropriate prescribing in older people: version 2. Age Ageing.

[15] Brown JD, Hutchison LC, Li C, Painter JT, Martin BC (2016). Predictive validity of the beers and screening tool of older persons’ potentially inappropriate prescriptions (STOPP) criteria to detect adverse drug events, hospitalizations, and emergency department visits in the United States. J Am Geriatr Soc.

[16] Moriarty F, Bennett K, Kenny RA, Fahey T, Cahir C (2020). Comparing potentially inappropriate prescribing tools and their association with patient outcomes. J Am Geriatr Soc.

[17] Boland B, Guignard B, Dalleur O, Lang P-O (2016). Application of STOPP/START and Beers criteria: compared analysis on identification and relevance of potentially inappropriate prescriptions. Eur Geriatr Med.

[18] Thomas RE, Nguyen LT, Jackson D, Naugler C (2020). Potentially inappropriate prescribing and potential prescribing omissions in 82,935 older hospitalised adults: association with hospital readmission and mortality within six months. Geriatrics.

[19] Pulok MH, Theou O, van der Valk AM, Rockwood K (2020). The role of illness acuity on the association between frailty and mortality in emergency department patients referred to internal medicine. Age Ageing.

[20] Quan H, Li B, Couris CM, Fushimi K, Graham P, Hider P, Januel J-M, Sundararajan V (2011). Updating and validating the charlson comorbidity index and score for risk adjustment in hospital discharge abstracts using data from 6 countries. Am J Epidemiol.

[21] Muhlack DC, Hoppe LK, Saum K-U, Haefeli WE, Brenner H, Schöttker B (2020). Investigation of a possible association of potentially inappropriate medication for older adults and frailty in a prospective cohort study from Germany. Age Ageing.

[22] Tian F, Liao S, Chen Z, Xu T (2021). The prevalence and risk factors of potentially inappropriate medication use in older Chinese inpatients with multimorbidity and polypharmacy: a cross-sectional study. Ann Transl Med..

[23] Poudel A, Peel NM, Nissen L, Mitchell C, Gray LC, Hubbard RE (2014). Potentially inappropriate prescribing in older patients discharged from acute care hospitals to residential aged care facilities. Ann Pharmacother.

[24] Mucalo I, Hadžiabdić MO, Brajković A, Lukić S, Marić P, Marinović I, Bačić-Vrca V (2017). Potentially inappropriate medicines in elderly hospitalised patients according to the EU(7)-PIM list, STOPP version 2 criteria and comprehensive protocol. Eur J Clin Pharmacol.

[25] Hudhra K, Beçi E, Petrela E, Xhafaj D, García-Caballos M, Bueno-Cavanillas A (2016). Prevalence and factors associated with potentially inappropriate prescriptions among older patients at hospital discharge. J Eval Clin Pract.

[26] Buda V, Prelipcean A, Andor M, Dehelean L, Dalleur O, Buda S, Spatar L, Mabda MC, Suciu M, Danciu C, Tudor A, Petrescu L, Cristescu C (2020). Potentially inappropriate prescriptions in ambulatory elderly patients living in rural areas of Romania using STOPP/START (Version 2) criteria. CIA.

[27] Ryan C, O’Mahony D, Kennedy J, Weedle P, Byrne S (2009). Potentially inappropriate prescribing in an Irish elderly population in primary care. Br J Clin Pharmacol.

[28] Kotsani M, Ellul J, Bahat G, Bogdanovic N, Burazeri G, Erceg P, Petreska-Zovic B, Prada GI, Smyrnakis E, Veninšek G, Zamboulis C, Martin FC, Petrovic M, Benetos A (2020). Start low, go slow, but look far: the case of geriatric medicine in Balkan countries. Eur Geriatr Med.

[29] van Poelgeest EP, Seppala LJ, Lee JM, Bahat G, Ilhan B, Lavan AH, Mair A, van Marum RJ, Onder G, Ryg J, Fernandes MA, Garfinkel D, Guðmundsson A, Hartikainen S, Kotsani M, Montero-Errasquín B, Neumann-Podczaska A, Pazan F, Petrovic M, Soulis G, Vankova H, Wehling M, Wieczorowska-Tobis K, van der Velde N (2022). On Behalf of the EuGMS SIG Pharmacology Deprescribing practices, habits and attitudes of geriatricians and geriatricians-in-training across Europe: a large web-based survey. Eur Geriatr Med.

[30] McIntosh J, Alonso A, MacLure K, Stewart D, Kempen T, Mair A, Castel-Branco M, Codina C, Fernandez-Llimos F, Fleming G, Gennimata D, Gillespie U, Harrison C, Illario M, Junius-Walker U, Kampolis CF, Kardas P, Lewek P, Malva J, Menditto E, Scullin C, Wiese B, On behalf of the SIMPATHY Consortium (2018). A case study of polypharmacy management in nine European countries: implications for change management and implementation. PLoS One..

[31] Sayın Z, Sancar M, Özen Y, Okuyan B (2022). Polypharmacy, potentially inappropriate prescribing and medication complexity in Turkish older patients in the community pharmacy setting. Acta Clin Belg.

[32] Xu Z, Liang X, Zhu Y, Lu Y, Ye Y, Fang L, Qian Y (2021). Factors associated with potentially inappropriate prescriptions and barriers to medicines optimisation among older adults in primary care settings: a systematic review. Fam Med Com Health..

[33] Baré M, Lleal M, Ortonobes S, Gorgas MQ, Sevilla-Sánchez D, Carballo N, De Jaime E, Herranz S (2022). on behalf of the MoPIM study group, factors associated to potentially inappropriate prescribing in older patients according to STOPP/START criteria: MoPIM multicentre cohort study. BMC Geriatr.

[34] Shade MY, Berger AM, Chaperon C, Haynatzki G, Sobeski L, Yates B (2017). Factors associated with potentially inappropriate medication use in rural, community-dwelling older adults. J Gerontol Nurs.

[35] Nothelle SK, Sharma R, Oakes A, Jackson M, Segal JB (2019). Factors associated with potentially inappropriate medication use in community-dwelling older adults in the United States: a systematic review†. Int J Pharm Pract.

[36] Lin Y-J, Peng L-N, Chen L-K, Lin M-H, Hwang S-J (2011). Risk factors of potentially inappropriate medications among older patients visiting the community health center in rural Taiwan. Arch Gerontol Geriatr.

[37] Al-Ragawi AM, Zyryanov SK, Ushkalova EA, Butranova OI, Pereverzev AP (2020). Prevalence and risk factors of potentially prescribing omissions in elderly and senile patients: clinical practice in Russian hospitals. Adv Gerontol.

