# Supplementary material for: Inappropriate prescribing in geriatric rural primary care: impact on adverse outcomes and relevant risk factors in a prospective observational cohort study
Source: Aging Clin Exp Res. 2023 Jul 10;35(9):1901–7. doi: 10.1007/s40520-023-02475-y (PMC10460359; doi:10.1007/s40520-023-02475-y)
Supplement: Supplementary file 1 — Supplementary file1 (DOCX 65 KB) [file 40520_2023_2475_MOESM1_ESM.docx]

**Supplemental material**

**Title page**

**Inappropriate prescribing in geriatric rural primary care: impact on adverse outcomes and relevant risk factors in a prospective observational cohort study**

*Maria Tampaki^1^, Alexandra Livada^2^, Maria-Niki Fourka^2^, Elli Lazaridou^3^, Marina Kotsani^4^, Athanase Benetos^5^, Petros P Sfikakis^6^, and Evrydiki Kravvariti^6^*

1. Postgraduate Medical Studies in Geriatric Syndromes and Physiology of Aging, School of Medicine, National and Kapodistrian University of Athens, Athens, Greece
2. Department of Statistics, Athens University of Economics and Business, Greece
3. Primary medical care unit of Marmari, S. Evia, General Hospital of Karystos, Greece
4. Université de Lorraine, CHRU-Nancy, Pôle « Maladies du Vieillissement, Gérontologie et Soins Palliatifs », Nancy, France and Hellenic Society for the Study and Research of Aging, Athens, Greece
5. Université de Lorraine, CHRU-Nancy, Pôle « Maladies du Vieillissement, Gérontologie et Soins Palliatifs », and INSERM DCAC u1116, F-54000, Nancy, France
6. First department of propaedeutic and internal medicine, Joint Academic Rheumatology Program, School of Medicine, National and Kapodistrian University of Athens, Athens, Greece

**Corresponding Author**: Evrydiki Kravvariti

postal address: 75, Mikras Asias St., Goudi, Athens, 11527

email address: [ev.kravvariti@gmail.com](mailto:ev.kravvariti@gmail.com)

telephone number: +306936876850

Online Resource 1 Prevalence of most common PIMs

Online Resource 1: Among those with PIM the most frequent cause of inappropriateness was drug prescription beyond the recommended duration, followed by drug-prescription without an evidence-based clinical indication, duplicate drug class prescription and the prescription of drugs to treat side effects of other drugs.

Online Resource 2 Prevalence of most common PPOs

Online Resource 2: Among PPOs the vast majority regarded vaccination omission.

Online Resource 3 Correlations among quantitative variables used for multiple logistic regression models

|  | Age | CFS | GFR | Κatz Index | CCI | Nr of PIMS | Nr of meds |
| --- | --- | --- | --- | --- | --- | --- | --- |
| Age | 1 |  |  |  |  |  |  |
| CFS | 0.626* | 1 |  |  |  |  |  |
| GFR | 0.171* | 0.201* | 1 |  |  |  |  |
| Κatz Index | 0.452* | -0.532* | 0.229* | 1 |  |  |  |
| CCI | 0.251* | 0.311* | 0.117 | -0.246 | 1 |  |  |
| Nr of PIMS | 0.223* | 0.335* | 0.017 | -0.051 | 0.209* | 1 |  |
| Nr of meds | 0.254* | 0.362* | 0.033 | 0.198* | 0.624* | 0.545* | 1 |

To test correlations among quantitative variables used for multiple logistic regression models the non-parametric Spearman test was used. † Spearman's rho value is shown and p-values < 0.05 are signed with (*) ‡ Nr of meds: number of medications, CFS: Clinical frailty scale, GFR: glomerular filtration rate, Nr of PIMs: number of potentially inappropriate medications, Nr of meds: number of medications, CCI: Charlson comorbidity Index

Online Resource 4 Correlations of sex and other quantitative variables

|  |  | W | p-value |
| --- | --- | --- | --- |
| Sex | Age | 1467.5 | 0.452 |
|  | Clinical Frailty Scale | 1373.5 | 0.888 |
|  | katz index | 1203.5 | 0.145 |
|  | CCI | 1525 | 0.234 |
|  | Nr of PIMs | 908.5 | 0.002* |
|  | Nr of meds | 1150.5 | 0.190 |

To test correlations between sex and other quantitative variables non-parametric Wilcoxon test was used. †W values with corresponding p-values of the controls are shown. p-value < 0.05 are signed with (*) ‡ Nr of meds: number of medications, Nr of PIMs: number of potentially inappropriate medications, Nr of meds: number of medications, CCI: Charlson comorbidity Index

Online Resource 5 Sample characteristics among PIM and no PIM

Online Resource 5: PIM is more prevalent among older people, women and frail patients, †CCI: Charlson Comorbidity Index, CFS: Clinical Frailty Scale, PIM: potentially inappropriate medications

Online Resource 6 Further data of the whole sample, PIMs and acute care visits regarding CCI, CFS and female sex

|  | Overall | PIM | | 6-month acute care visit | |
| --- | --- | --- | --- | --- | --- |
|  |  | Yes | No | Yes | No |
| CCI (median, IQR) | 1 (0-1) | 1(0-2) | 1 (0-1) | 1(0-2) | 1(0-1) |
| CFS (median, IQR) | 4 (1-7) | 4(3-5) | 2(2-4) | 4(3-5) | 3(2-4) |
| Female % | 49.1% | 57,1% | 34.1% | 54.8% | 46.6% |

Online Resource 6 PIM is more prevalent among women and frail patients, †CCI: Charlson Comorbidity Index, CFS: Clinical Frailty Scale, IQR: interquartile range, 6-month acute care visit: the composite of emergency department visits and/or hospitalization
